# Perceived teacher–student power distance in relation to campus connectedness profiles among Chinese college students: profile differences in difficulties in emotion regulation

**DOI:** 10.3389/fpsyg.2026.1906678

**Published:** 2026-08-27

**Authors:** Jiawei Xing, Yuchen Shen, Juan Li, Yuheng Zhang

**Affiliations:** 1 Educational Faculty, Jiangxi Science & Technology Normal University, Nanchang, China; 2 School of Health Care Security, Shandong First Medical University, Jinan, China

**Keywords:** campus connectedness, difficulties in emotion regulation, latent profile analysis, perceived teacher–student power distance, social belonging

## Abstract

**Introduction:**

As a core expression of social belonging within the campus community, campus connectedness can be regarded as a relational foundation of student flourishing, but has typically been examined as a continuous construct using variable-centered approaches, leaving its person-level patterning less well understood. Adopting a person-centered perspective, this study identified latent profiles of campus connectedness. Given the cultural and institutional salience of teacher–student power distance in Chinese higher education, the association between students' perceived teacher–student power distance and profile membership was further examined. Difficulties in emotion regulation were also compared across profiles to assess the external validity and psychological relevance of the profile solution.

**Methods:**

A sample of 3,885 Chinese college students completed measures of campus connectedness, perceived teacher–student power distance, and difficulties in emotion regulation. Latent profile analysis was conducted using the Campus Connectedness Scale items. R3STEP examined associations between perceived power distance and profile membership, and BCH compared difficulties in emotion regulation across profiles.

**Results:**

Three ordered, level-based profiles were retained, comprising low (9.1%), moderate (53.6%), and high (37.3%) campus connectedness. Higher perceived teacher-student power distance was associated with greater odds of low- versus high-connectedness membership (OR = 1.347, *p* < .001) and moderate- versus high-connectedness membership (OR = 1.449, *p* < .001), whereas the low-moderate contrast was weaker and model-dependent. Difficulties in emotion regulation decreased monotonically across the low-, moderate-, and high-connectedness profiles (M = 3.706, 3.374, and 2.522, respectively), with all pairwise differences significant at *p* < .001.

**Discussion:**

The association between perceived power distance and profile membership exhibited an asymmetric rather than a uniformly graded pattern, which may be consistent with the cultural and institutional normalization of teacher-student hierarchy in Chinese higher education. Furthermore, the monotonic decrease in difficulties in emotion regulation not only supported the external validity of the profile solution but also indicated that differences in students' campus-specific social belonging co-occurred with meaningful differences in emotional adjustment relevant to resilient functioning.

## 1 Introduction

Higher education institutions function not merely as academic settings but also as social environments in which students cultivate relationships, construct their identities, and give and receive social support, making campus connectedness an increasingly important focus of scholarly attention (Fiset and Robertson, 2023; Bond et al., 2026; Doan et al., 2026). As a campus-specific form of social connectedness (Lee and Davis, 2000; Lee and Yoo, 2002), campus connectedness reflects students' subjective experience of their relational standing within the campus community, representing a core expression of social belonging in the campus context (Lee and Robbins, 1995; Allen et al., 2021; Dias-Broens et al., 2024). It encompasses students' institutional identification and their perceived acceptance, closeness, and relational integration with peers, faculty, staff, and the campus community as a whole (Summers et al., 2005; Wong et al., 2019; Jouriles et al., 2020; Johnson and Plisco, 2025; Navarro Silvera et al., 2026). Because social belonging constitutes the relational dimension of student wellbeing (Zhao and Hua, 2025) and further predicts students' mental health within the college environment (Walton and Cohen, 2007, 2011; Fink, 2014), campus connectedness can be regarded as a relational foundation of student flourishing (Keyes, 2002). Consistent with this view, previous studies have linked campus connectedness to lower psychological distress (Adams et al., 2021; Di Malta et al., 2022; Johnson and Plisco, 2025; Navarro Silvera et al., 2026) and more positive psychological functioning (Di Malta et al., 2022; Diggs et al., 2025; Karadag et al., 2025).

However, most previous research has examined campus connectedness as a continuous construct, focusing primarily on overall levels and associations with other variables, while its person-level patterning remains less understood. A person-centered approach offers a complementary perspective by examining whether latent profiles can be identified across multiple indicators (Marsh et al., 2009; Spurk et al., 2020). Beyond profile identification, person-centered analysis also enables profile-level external association tests, providing information on whether and to what extent external variables differentiate membership across the retained profile contrasts, thereby complementing population-level associations estimated using continuous scores (Morin and Marsh, 2015; Spurk et al., 2020). To date, no study has examined campus connectedness using a person-centered framework that integrates profile identification with profile-level external association tests. In the broader social connectedness literature, prior person-centered work has occasionally reported different patterns (Rose et al., 2019; Teas et al., 2023; Oberle et al., 2024), but whether similar patterns characterize campus connectedness as a campus-specific manifestation of social connectedness remains unclear. Accordingly, a person-centered examination of campus connectedness may extend existing research by describing its person-level patterning.

Moreover, perceived power distance in teacher–student relationships may be concurrently associated with campus connectedness profile membership among Chinese college students. For college students, higher education institutions function not only as communities of peers (Sollitto et al., 2013; Wilson and Gore, 2013; Thelamour et al., 2019), but also as hierarchical social organizations in which students interact with institutional authorities (Symonds, 2021). This hierarchy is particularly salient in Chinese higher education, where it is embedded in the Confucian educational tradition and reinforced by structural features of the system (Dai and Matthews, 2023; Matthews and Dollinger, 2023; Liang et al., 2024). Faculty members are the most immediately encountered actors within this hierarchy, because they maintain frequent contact with students while holding instructional, evaluative, and advisory authority (Hagenauer and Volet, 2014; Xu and Zhang, 2025). Accordingly, students' perceptions of power distance in teacher–student relationships may represent a proximal way in which institutional hierarchy is experienced on campus (Zhang, M., et al., 2025). Prior research has linked faculty-related experiences to students' sense of belonging on campus, such as student–faculty interaction and faculty support (Kirby and Thomas, 2022; Abdulhamed and Beattie, 2024; Kim and Lundberg, 2026). Given that campus connectedness represents the sense of belonging in its campus-specific form (Lee and Robbins, 1995; Allen et al., 2021; Dias-Broens et al., 2024), these findings may extend to students' connectedness to the campus community. However, little is known about whether students' perceived power distance in teacher–student relationships is associated with membership in distinct campus connectedness profiles.

Beyond examining the concurrent association between perceived power distance and profile membership, it is also important to assess profile-level differences in psychological functioning (Asparouhov and Muthen, 2014; Dziak et al., 2016). Difficulties in emotion regulation (DER) provide a meaningful indicator of constrained adaptive functioning among college students. DER generally refers to difficulties in emotional awareness, clarity, and acceptance, as well as problems maintaining goal-directed behavior, controlling impulses, and flexibly using effective regulation strategies under negative emotional states (Gratz and Roemer, 2004). The college period involves multiple challenges related to academic demands, interpersonal relationships, identity development, and future planning, requiring students to cope continuously with stress and negative emotions (Lin and Huang, 2014; Li et al., 2022). In this context, students' ability to regulate emotions is closely related to their psychological resilience (Polizzi and Lynn, 2021; Stover et al., 2024), defined as the capacity to adapt successfully to challenges that threaten adaptive functioning (Masten et al., 2021). Elevated DER may therefore be understood not simply as a correlate of distress but as a constraint on the capacities that support students' resilient adjustment to campus life. Prior research has documented associations between stronger social connectedness and better emotional functioning (Xu et al., 2024; Huang et al., 2025). As a specific form of social connectedness (Lee and Davis, 2000; Lee and Yoo, 2002), campus connectedness may likewise be negatively associated with DER. Given that social connectedness can be organized into distinct profiles (Rose et al., 2019; Teas et al., 2023; Oberle et al., 2024), campus connectedness profiles may similarly correspond to different levels of DER, providing evidence regarding the external validity and psychological relevance of the profile solution.

Based on the above considerations, the present study aimed to adopt a person-centered approach to examine profiles of campus connectedness among college students. The study further examined whether students' perceived power distance in teacher–student relationships was concurrently associated with membership in these profiles, thereby clarifying how perceived institutional hierarchy may relate to different patterns of campus connectedness. Finally, the study investigated whether the identified profiles differed in difficulties in emotion regulation, a constraint on the adaptive functioning and resilience that underpin flourishing during the college years. By adopting a person-centered framework, the present study was intended to complement previous variable-centered research by examining the person-level representation of campus connectedness and its profile-level associations with perceived institutional hierarchy and difficulties in emotion regulation.

## 2 Materials and methods

### 2.1 Participants

Data were collected using a combination of convenience sampling and snowball sampling. The researchers first contacted university administrative staff, student counselors, and course instructors, explained the study purpose, questionnaire content, participation principles, and data confidentiality requirements, and invited them to disseminate the electronic questionnaire link to undergraduate students. On this basis, some initial contacts further invited other student counselors or course instructors to assist in distributing the questionnaire link, thereby broadening the sample coverage. All participants completed the questionnaire anonymously and voluntarily, and were informed of the study purpose and their right to withdraw before participation. The study was approved by the Institutional Review Board of the Jiangxi Psychological Counselor Association (IRB No. JXSXL-2024-SEP013), complied with the Declaration of Helsinki, and included only adult participants who provided informed consent.

A total of 3,885 valid participants (mean age = 19.5 years, SD = 1.37) were included in the present study. Of these, 1,076 were male (27.7%) and 2,809 were female (72.3%). In terms of age, 1,011 participants were 18 years old (26.0%), 1,175 were 19 years old (30.2%), 929 were 20 years old (23.9%), 455 were 21 years old (11.7%), and 315 were 22 years old or above (8.1%). Regarding academic year, 1,756 participants were first-year college students (45.2%), 1,117 were second-year students (28.8%), 851 were third-year students (21.9%), and 161 were fourth-year students (4.1%). With respect to institution type, 2,579 participants were enrolled in undergraduate institutions (66.4%), whereas 1,306 were enrolled in junior colleges or vocational colleges (33.6%). Participants' monthly household income (MHI) was distributed as follows: 799 participants reported an MHI of 0–2,000 RMB (20.6%), 1,292 reported 2,001–4,000 RMB (33.3%), 851 reported 4,001–6,000 RMB (21.9%), 449 reported 6,001–8,000 RMB (11.6%), 252 reported 8,001–10,000 RMB (6.5%), and 242 reported more than 10,000 RMB (6.2%).

### 2.2 Measures

#### 2.2.1 Campus Connectedness Scale

The Campus Connectedness Scale (CCS), developed by Lee and Robbins (1995) and revised by Lee and Yoo (2002), was used in the present study. The scale was designed to assess students' perceived connectedness within the overall campus environment. The CCS comprises 14 items and is treated as a unidimensional measure. A sample item is “There are people on campus with whom I feel a close bond”. All items are rated on a 7-point Likert scale ranging from 1 (strongly disagree) to 7 (strongly agree), with higher scores indicating stronger perceived campus connectedness. The CCS has previously been used among Chinese college students and demonstrated satisfactory internal consistency (Cronbach's α = 0.86; Gong et al., 2022). In the present study, Cronbach's α for the scale was 0.96.

#### 2.2.2 Power distance scale

Perceived teacher–student power distance was measured using a scale adapted from the Chinese version (Meng, 2019) of Dorfman and Howell's Power Distance Scale (Dorfman and Howell, 1988), with item wording revised to fit the teacher–student context and to capture students' perceptions of existing power distance. The original scale was designed to assess individuals' acceptance of unequal power distribution and the use of authority in organizational settings. In the present study, the original superior–subordinate wording was adapted to the teacher–student context to assess college students' perceptions of power distance in teacher–student relationships. A sample item is “I think it is difficult for students to oppose their teachers' decisions.” All items are rated on a 7-point Likert scale ranging from 1 (strongly disagree) to 7 (strongly agree), with higher scores indicating greater perceived teacher–student power distance. The original scale has previously demonstrated satisfactory internal consistency in Chinese subjects (Cronbach's α = 0.78–0.88; Bao et al., 2019; Zhang, J., et al., 2025). In the present sample of Chinese college students, Cronbach's α for the adapted scale was 0.91.

#### 2.2.3 Difficulties in Emotion Regulation Scale Short Form

The Difficulties in Emotion Regulation Scale Short Form (DERS-SF), developed by Kaufman et al. (2016), is a shortened version of the original Difficulties in Emotion Regulation Scale (DERS) developed by Gratz and Roemer (2004). The scale assesses difficulties in emotion regulation across six subscales: Non-acceptance, referring to non-acceptance of emotional responses; Goals, referring to difficulties engaging in goal-directed behavior; Impulse, referring to impulse control difficulties; Awareness, referring to lack of emotional awareness; Strategies, referring to limited access to emotion regulation strategies; and Clarity, referring to lack of emotional clarity. Sample items include “When I'm upset, I have difficulty focusing on other things” and “When I'm upset, I have difficulty controlling my behavior.” The scale consists of 18 items rated on a 7-point Likert scale ranging from 1 (strongly disagree) to 7 (strongly agree), with higher scores indicating greater difficulties in emotion regulation. The DERS-SF has previously been used among Chinese student samples (Cronbach's α = 0.89–0.93; Jiang et al., 2022; Li et al., 2024; Wang et al., 2026). In the present study, the Cronbach's α coefficient of this scale was 0.96.

### 2.3 Statistical analysis

Descriptive statistics, profile-specific demographic distributions, and Pearson correlations among campus connectedness, perceived teacher–student power distance, and difficulties in emotion regulation were computed using IBM SPSS Statistics 25.0. All model-based analyses were conducted using Mplus 8.3. Figures were generated using Microsoft Excel 2019. Confirmatory factor analysis was first performed to examine the measurement properties of the study instruments. The Campus Connectedness Scale and the Power Distance Scale were each specified as a unidimensional model, and the Difficulties in Emotion Regulation Scale Short Form was specified as a second-order factor model, with 18 items loading onto six first-order dimensions and the six dimensions loading onto a higher-order DER factor. Model fit was evaluated using RMSEA, SRMR, CFI, and TLI.

Latent profile analysis was then conducted using the 14 items of the Campus Connectedness Scale as profile indicators. Models with one to six profiles were estimated and compared using maximum likelihood estimation with robust standard errors (MLR). The optimal solution was selected based on AIC, BIC, aBIC, entropy, LMRT, BLRT, profile size, substantive interpretability, and parsimony. Lower AIC, BIC, and aBIC values indicated better fit, and higher entropy values indicated greater classification precision. Based on these criteria, the two-profile and six-profile solutions were excluded, and the three-, four-, and five-profile solutions were retained for further comparison.

To determine whether the four- and five-profile solutions represented qualitatively new configurations or primarily subdivided profiles identified in the three-profile solution, class-specific item means from the three-, four-, and five-profile solutions were standardized using the corresponding full-sample item means and standard deviations. The 14 standardized item means within each profile were then centered by subtracting their within-profile mean. Centered Tucker's congruence coefficients were then calculated between profiles from the three-profile solution and those from the four- and five-profile solutions, with coefficients closer to 1 indicating greater similarity in profile shape. Profile elevation was calculated as the mean of the 14 standardized item means within each profile. Standardized profile plots were generated to facilitate visual comparisons of profile elevation and shape across the competing solutions. As a robustness check, the one- through six-profile models were re-estimated separately in the female and male subsamples using the same indicators, model specifications, and selection criteria as in the full sample. Cross-sex similarity of the retained three-profile solution was assessed using centered Tucker's congruence coefficients based on pooled-sample standardized item means, with profile elevations and proportions compared descriptively.

After the optimal profile solution was identified, the demographic composition of each profile was summarized using frequencies and within-profile percentages based on participants' most likely profile membership. To evaluate omnibus demographic differences across profiles while accounting for classification uncertainty, continuous age was examined using the Bolck–Croon–Hagenaars (BCH) procedure, whereas sex, academic year, institution type, and monthly household income were examined using the categorical distal outcome (DCAT) procedure. Omnibus differences were evaluated using Wald chi-square tests.

The R3STEP procedure was then used to estimate the concurrent association between perceived teacher–student power distance and profile membership while accounting for classification uncertainty. As a sensitivity check, the R3STEP analysis was re-estimated with sex, age, institution type, academic year, and monthly household income included as covariates. To complement the profile-based analyses, a continuous-score analysis examined linear and quadratic associations between perceived teacher–student power distance and campus connectedness. Finally, the BCH method was used to compare difficulties in emotion regulation across the identified profiles, with omnibus and pairwise chi-square tests used to evaluate profile differences. Because perceived power distance and campus connectedness were measured at the same assessment occasion, the resulting coefficients and odds ratios were interpreted as associational estimates and do not establish temporal precedence, directionality, or causality.

## 3 Results

### 3.1 Descriptive statistics and bivariate correlations

Table 1 shows the descriptive statistics and correlation analysis results for the measures of Campus Connectedness, Power Distance, and Difficulties in Emotion Regulation. The results indicated that campus connectedness was significantly negatively correlated with power distance and difficulties in emotion regulation. Power distance was significantly positively correlated with difficulties in emotion regulation.

**Table 1:** Descriptive statistics and pearson correlation.

Construct	Mean	SD	1	2	3
1.CC	4.94	1.12	1		
2.PD	3.34	1.33	−0.177^**^	1	
3.DER	3.08	1.32	−0.341^**^	0.313^**^	1

SD, Standard Deviation; CC, Campus Connectedness; PD, Power Distance; DER, Difficulties in Emotion Regulation. ^**^ p < 0.01

.

### 3.2 CFA

Before conducting the main analyses, confirmatory factor analysis was performed to examine the measurement properties of the study instruments. For the DERS-SF, a second-order factor structure was specified, with the 18 items loading onto six first-order dimensions and the six dimensions loading onto a higher-order DER factor. The measurement model showed an acceptable fit to the data, with RMSEA = 0.048, SRMR = 0.041, CFI = 0.933, and TLI = 0.929. The standardized factor loadings are presented in Table 2. The standardized loadings ranged from 0.604 to 0.851 for the Campus Connectedness items and from 0.725 to 0.875 for the Power Distance items. For Difficulties in Emotion Regulation, both the item-level loadings on their corresponding first-order dimensions and the first-order dimension loadings on the higher-order DER factor were within acceptable ranges. In addition, all estimated unstandardized coefficients were statistically significant at *p* < 0.001.

**Table 2:** Factor loadings and significance tests from CFA.

Item	Std.	Unstd.	S.E.	Std./S.E.	*p*-Value
CC1	0.604	1.000	-	-	-
CC2	0.742	1.075	0.025	43.098	< 0.001
CC3	0.725	1.109	0.026	42.138	< 0.001
CC4	0.711	1.170	0.031	37.858	< 0.001
CC5	0.796	1.173	0.027	42.903	< 0.001
CC6	0.798	1.180	0.029	40.474	< 0.001
CC7	0.795	1.102	0.029	37.578	< 0.001
CC8	0.825	1.169	0.030	39.446	< 0.001
CC9	0.770	1.075	0.031	34.536	< 0.001
CC10	0.823	1.392	0.035	39.910	< 0.001
CC11	0.851	1.276	0.033	39.148	< 0.001
CC12	0.826	1.275	0.033	38.637	< 0.001
CC13	0.822	1.177	0.032	36.939	< 0.001
CC14	0.851	1.300	0.033	39.877	< 0.001
PD1	0.794	1.000	-	-	-
PD2	0.725	0.963	0.019	51.403	< 0.001
PD3	0.875	1.062	0.015	69.830	< 0.001
PD4	0.766	0.999	0.018	55.791	< 0.001
PD5	0.865	1.077	0.016	66.600	< 0.001
PD6	0.788	0.920	0.018	51.092	< 0.001
DER1	0.773	1.000	-	-	-
DER2	0.828	1.038	0.018	58.650	< 0.001
DER3	0.864	1.166	0.019	61.578	< 0.001
DER4	0.876	1.000		-	-
DER5	0.900	1.039	0.012	89.329	< 0.001
DER6	0.852	1.022	0.014	74.676	< 0.001
DER7	0.892	1.000	-	-	-
DER8	0.932	1.016	0.011	90.660	< 0.001
DER9	0.901	1.027	0.012	88.829	< 0.001
DER10	0.926	1.000	-	-	-
DER11	0.958	1.025	0.008	131.539	< 0.001
DER12	0.884	0.919	0.010	92.928	< 0.001
DER13	0.870	1.000	-	-	-
DER14	0.769	0.979	0.017	57.013	< 0.001
DER15	0.859	0.999	0.016	63.413	< 0.001
DER16	0.857	1.000	-	-	-
DER17	0.932	1.094	0.012	88.317	< 0.001
DER18	0.881	1.011	0.015	69.559	< 0.001
DERI	0.908	1.000	-	-	-
DERII	0.911	1.109	0.021	53.784	< 0.001
DERIII	0.823	0.975	0.020	48.864	< 0.001
DERIV	0.753	1.082	0.022	49.846	< 0.001
DERV	0.775	0.836	0.021	40.632	< 0.001
DERVI	0.845	1.024	0.021	48.750	< 0.001

Std., Standardized coefficient; Unstd., Unstandardized coefficient; S.E., Standard Error; CC1–CC14, Items of Campus Connectedness; PD1–PD6, items of Power Distance; DER1–DER18, Items of Difficulties in Emotion Regulation; DERI–DERVI, six dimensions of DER, with DERI including DER1–DER3, DERII including DER4–DER6, DERIII including DER7–DER9, DERIV including DER10–DER12, DERV including DER13–DER15, and DERVI including DER16–DER18.

### 3.3 Campus connectedness profile solution

**Figure 1:**
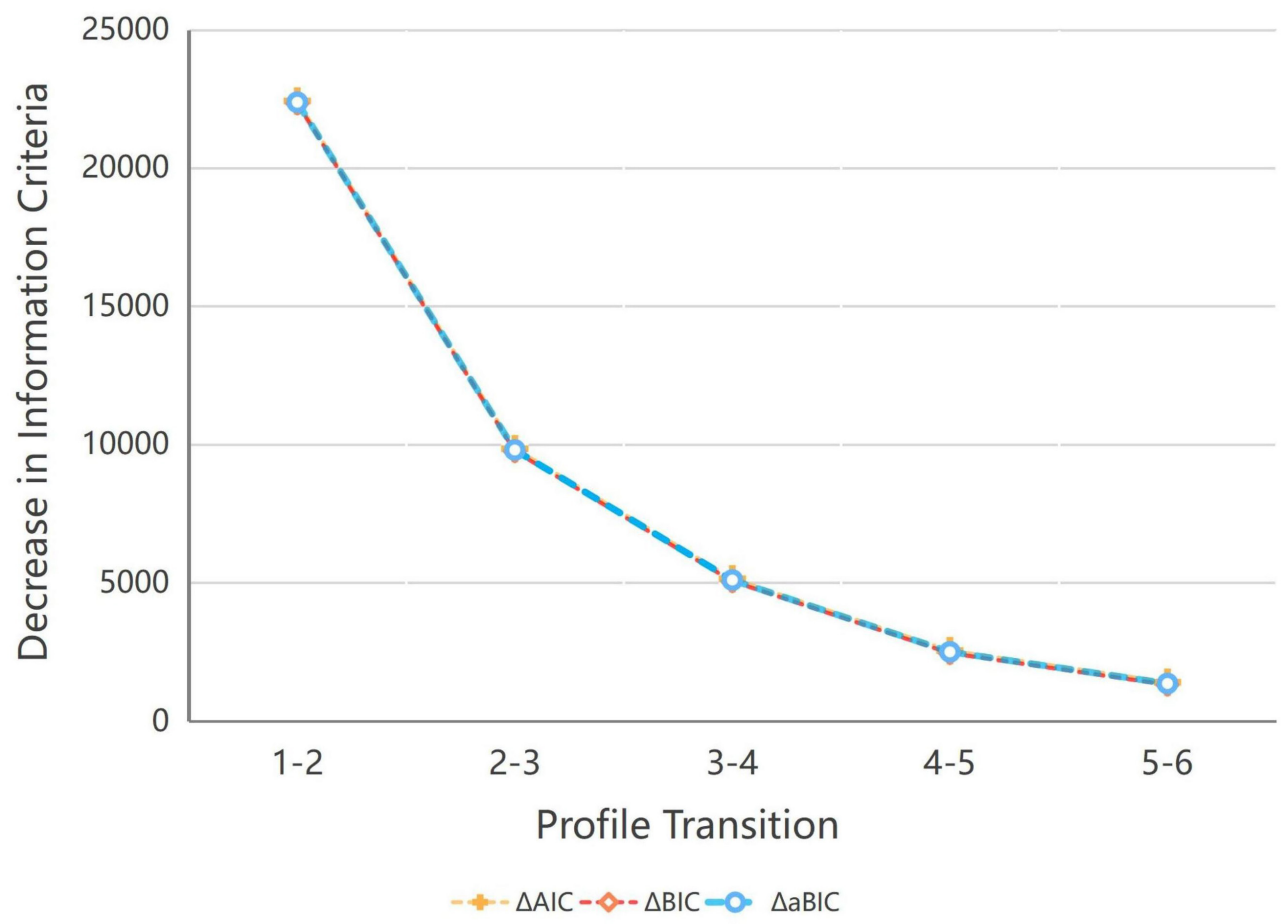
Changes in information criteria indices across profile solutions.

As shown in Table 3, the AIC, BIC, and aBIC decreased as the number of profiles increased from one to six. For the two- through six-profile solutions, all entropy values exceeded 0.90, indicating good classification accuracy. In addition, both the LMRT and BLRT were significant for all tested solutions. The substantial decreases in AIC, BIC, and aBIC from the two-profile to the three-profile solution, together with the higher entropy of the three-profile solution (0.952 vs.0.925), favored the three-profile solution over the two-profile solution. Accordingly, the two-profile solution was not retained. Thereafter, the decreases in the information criterion values became progressively smaller, with the smallest decreases occurring from the five-profile to the six-profile solution, as illustrated in Figure 1. Furthermore, the smallest profile in the six-profile solution accounted for only 2.8% of the sample, raising concerns about its stability and substantive interpretability. Therefore, the six-profile solution was also excluded from further consideration.

**Table 3:** Model fit indices for profile solutions.

PN	AIC	ΔAIC	BIC	ΔBIC	aBIC	ΔaBIC	ENTROPY	LMRT	BLRT	PMG
P1	191137.3	-	191312.7	-	191223.7	-	-	-	-	-
P2	168712.5	22424.8	168981.8	22330.9	168845.2	22378.5	0.925	< 0.001	< 0.001	40.9%
**P3**	**158862.7**	**9849.8**	**159226.0**	**9755.8**	**159041.7**	**9803.5**	**0.952**	**< 0.001**	**< 0.001**	**9.1%** ^**a**^
P4	153716.2	5146.4	154173.6	5052.4	153941.6	5100.1	0.928	< 0.001	< 0.001	5.3%
P5	151167.0	2549.2	151718.3	2455.2	151438.7	2502.9	0.925	< 0.001	< 0.001	5.0%
P6	149761.1	1405.9	150406.4	1311.9	150079.1	1359.6	0.924	0.010	< 0.001	2.8%

PN, Profile Number; AIC, Akaike Information Criterion; BIC, Bayesian Information Criterion; aBIC, Sample-size-adjusted BIC; LMRT, Lo-Mendell-Rubin Adjusted Likelihood Ratio Test; BLRT, Bootstrapped Likelihood Ratio Test; PMG, Proportion of Minimum Group.

^a^The selected solution is indicated in bold.

**Figure 2:**
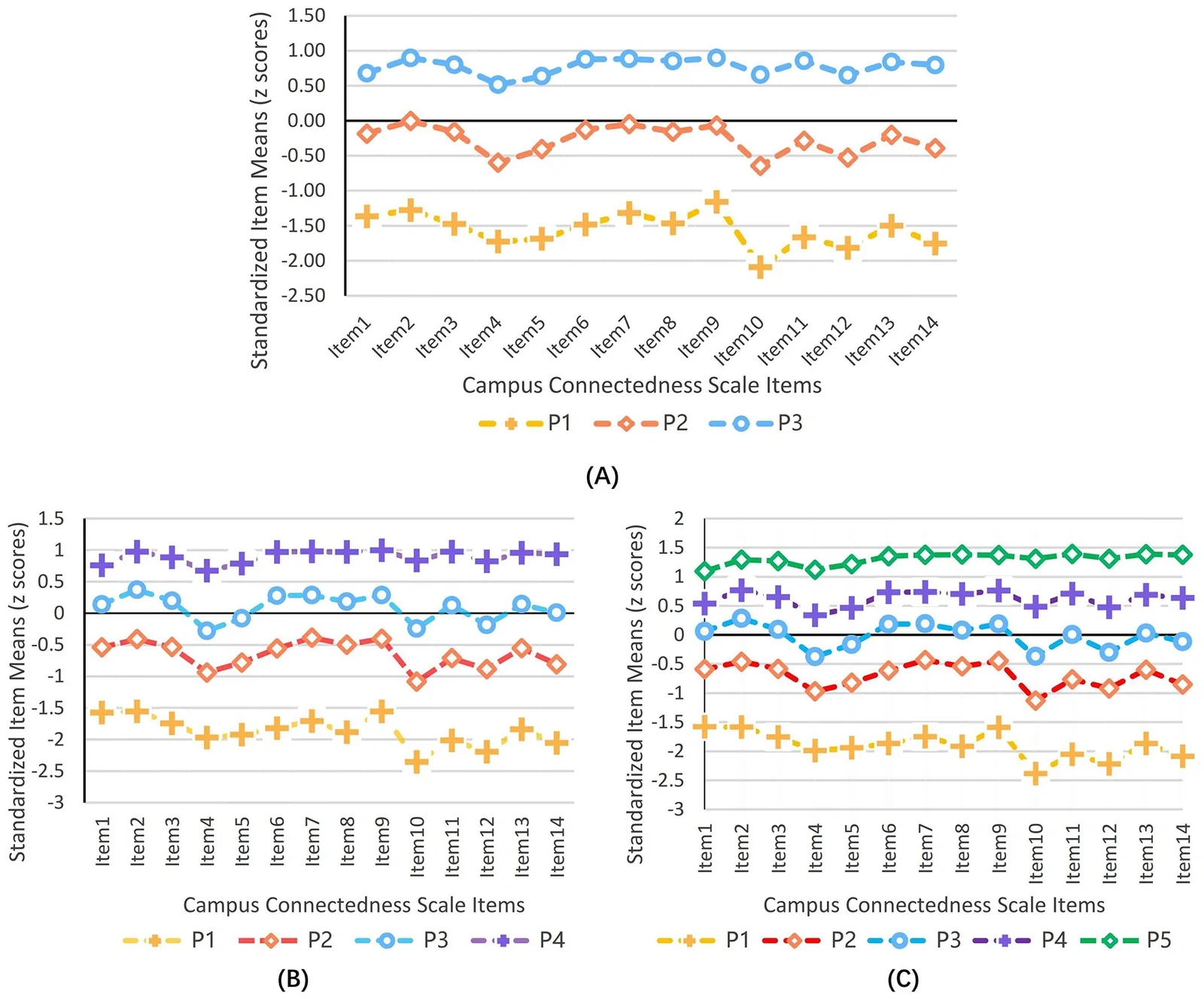
Standardized Campus Connectedness Item Means Across the Three-, Four-, and Five-Profile Solutions. **(A-C)** Present the three-profile, four-profile, and five-profile solutions, respectively. Class-specific item means were standardized using the corresponding full-sample item means and standard deviations.

Among the remaining three-, four-, and five-profile solutions, the three-profile solution yielded the highest entropy value (0.952) and a larger minimum profile proportion (9.1%) than the four- and five-profile solutions. The latter solutions had lower entropy values of 0.928 and 0.925, respectively, and minimum profile proportions close to 5%, raising some concerns regarding profile stability and substantive interpretability. Moreover, the centered Tucker's congruence coefficients reported in Table 4 indicated that the additional profiles in the four- and five-profile solutions provided limited substantive or theoretical increment. Specifically, the four-profile solution primarily subdivided the moderate-connectedness profile, whereas the four lower-elevation profiles in the five-profile solution closely corresponded to profiles already identified in the three-profile solution, with their strongest congruence coefficients ranging from 0.953 to 0.998. Although the highest-elevation profile in the five-profile solution showed comparatively weaker shape correspondence with the high-connectedness profile in the three-profile solution, Figure 2 indicated that the four- and five-profile solutions, like the three-profile solution, were characterized primarily by ordered differences in overall campus connectedness rather than by qualitatively distinct configurational patterns. Considering classification quality, profile size, substantive interpretability, and parsimony, the three-profile solution was retained.

**Table 4:** Centered Tucker's congruence coefficients and profile elevations across the three-, four-, and five-profile solutions.

Panel A. Three- and four-profile solutions						
Profile	4P-1	4P-2	4P-3	4P-4	3P Elevation	
**3P-1**	**0.964**	0.961	0.872	0.482	−1.556	
**3P-2**	0.866	**0.985**	**0.988**	0.700	−0.272	
**3P-3**	0.502	0.780	0.898	**0.970**	0.774	
**4P Elevation**	−1.870	−0.650	0.087	0.893	-	
**Panel B. Three- and five-profile solutions**.						
Profile	5P-1	5P-2	5P-3	5P-4	5P-5	3P Elevation
**3P-1**	**0.953**	0.963	0.892	0.681	0.085	−1.556
**3P-2**	0.848	**0.981**	**0.993**	0.868	0.318	−0.272
**3P-3**	0.467	0.771	0.870	**0.998**	**0.752**	0.774
**5P Elevation**	−1.899	−0.696	−0.016	0.619	1.303	-

Congruence coefficient entries are centered Tucker's congruence coefficients calculated from within-profile mean-centered standardized item means. 3P, 4P, and 5P denote the three-, four-, and five-profile solutions, respectively, and the number following the hyphen identifies the profile within each solution. Profile elevation was calculated as the mean of the 14 standardized item means within each profile. Profiles within each solution are ordered by increasing elevation. Boldface indicates the strongest correspondence for each profile in the four- or five-profile solution.

Focusing on the retained solution, Panel A of Figure 2 shows that the three profiles were differentiated primarily by their overall levels of campus connectedness. Profile 1 (P1) accounted for 9.1% of the sample and exhibited the lowest standardized item means. Profile 2 (P2) accounted for 53.6% and exhibited intermediate means. Profile 3 (P3) accounted for 37.3% and exhibited the highest means. Accordingly, the three profiles were labeled the low-, moderate-, and high-connectedness profiles, respectively. Despite these level differences, the profiles displayed broadly similar configurations across the 14 indicators. The most notable localized variation involved Item 10, “There is a sense of brother/sisterhood with my college friends,” which occupied a relatively lower position in the lower-connectedness profiles. Supplementary material 1 showed that the gap between Item 10 and the mean of the remaining 13 CCS items widened as overall connectedness decreased. Sex-stratified analyses in Supplementary material 2 further indicated that the ordered three-profile structure was broadly reproducible across sex, despite modest differences in profile-shape correspondence and profile prevalence. As shown in Table 5, omnibus tests indicated that the profiles differed significantly in sex distribution and monthly household income distribution, but not in mean age, academic-year distribution, or institution-type distribution. Descriptively, students from lower-income households were relatively more prevalent in the low-connectedness profile.

**Table 5:** Demographic characteristics and omnibus comparisons across the three campus connectedness profiles.

Variables	Variable labels	P1 (*n* = 354)	P2 (*n* = 2,083)	P3 (*n* = 1,448)	χ^2^	*p*
**Sex**	Male	105(29.7%)	499(24.0%)	472(32.6%)	32.94	< 0.001
	Female	249(70.3%)	1584(76.0%)	976(67.4%)		
**Age, 19.5 (1.37)**	18	72(20.3%)	566(27.2%)	373(25.8%)	4.19	0.123
	19	125(35.3%)	601(28.9%)	449(31.0%)		
	20	69(19.5%)	503(24.1%)	357(24.7%)		
	21	55(15.5%)	246(11.8%)	154(10.6%)		
	≥ 22	33(9.3%)	167(8.0%)	115(7.9%)		
**Academic year**	First year	151(42.7%)	951(45.7%)	654(45.2%)	10.65	0.223
	Second year	102(28.8%)	585(28.1%)	430(29.7%)		
	Third year	82(23.2%)	470(22.6%)	299(20.6%)		
	Fourth year	19(5.4%)	77(3.7%)	65(4.5%)		
**Institution type**	Undergraduate institutions	244(68.9%)	1397(67.1%)	938(64.8%)	4.86	0.561
	Junior or vocational colleges	110(31.1%)	686(32.9%)	510(35.2%)		
**Monthly household income (RMB)**	¥ 0-2000	89(25.1%)	473(22.7%)	237(16.4%)	66.14	< 0.001
	¥ 2001-4000	126(35.6%)	702(33.7%)	464(32.0%)		
	¥ 4001-6000	81(22.9%)	447(21.5%)	323(22.3%)		
	¥ 6001-8000	35(9.9%)	223(10.7%)	191(13.2%)		
	¥ 8001-10000	12(3.4%)	128(6.1%)	112(7.7%)		
	> ¥ 10000	11(3.1%)	110(5.3%)	121(8.4%)		

Values are presented as n (column %) unless otherwise indicated. Frequencies and percentages were calculated within profiles based on participants' most likely profile membership. Age is presented as M (SD). Omnibus comparisons were conducted in Mplus using the BCH procedure for continuous age and the DCAT procedure for categorical demographic variables, accounting for uncertainty in profile classification. P1, low campus connectedness profile; P2, moderate campus connectedness profile; P3, high campus connectedness profile; RMB, renminbi.

### 3.4 Power distance and latent profile membership

The R3STEP approach was used to examine the association between perceived teacher–student power distance and campus connectedness profile membership. The results are presented in Table 6. In the unadjusted model, higher perceived power distance was associated with greater odds of membership in P1 rather than P3 (OR = 1.347, *p* < 0.001) and in P2 rather than P3 (OR = 1.449, *p* < 0.001). By contrast, the association with P2 rather than P1 membership was smaller and statistically nonsignificant (OR = 1.076, *p* = 0.087). Thus, statistically significant associations were observed for the two contrasts involving P3, but not for the P2-vs.-P1 contrast.

**Table 6:** R3STEP results for power distance across pairwise profile comparisons.

Comparison	*B*	S.E.	*p*-value	OR
P1 vs. P3	0.298	0.051	< 0.001	1.347
P2 vs. P3	0.371	0.036	< 0.001	1.449
P2 vs. P1	0.073	0.043	0.087	1.076

P1, low campus connectedness profile; P2, moderate campus connectedness profile; P3, high campus connectedness profile. B, logit regression coefficient; S.E., standard error; OR, odds ratio.

### 3.5 Sensitivity analysis

As shown in Table 7, the R3STEP analysis was re-estimated with demographic covariates (sex, age, institution type, academic year, and monthly household income) included as a sensitivity check. After adjustment, higher perceived power distance remained associated with greater odds of P1 rather than P3 membership and of P2 rather than P3 membership. These two comparisons yielded the largest odds-ratio contrasts (P1 vs. P3, OR = 1.328; P2 vs. P3, OR = 1.466). The distinction between the low and moderate profiles, which was non-significant in the main unadjusted model, reached statistical significance after adjustment (P2 vs P1, OR = 1.104, *p* = 0.027). Despite this change in the significance of the low–moderate distinction, the two models yielded a consistent overall pattern. That is, the association between perceived power distance and profile membership was strongest for comparisons involving the high-connectedness profile and weakest for the P2-vs.-P1 comparison. Additionally, as shown in Supplementary material 3, the significant quadratic term (*B* = 0.154, SE = 0.007, *p* < 0.001) increased the explained variance from 3.1% to 13.6% (Δ*R*^2^ = 0.104), indicating that the concurrent association between perceived teacher–student power distance and campus connectedness departed from linearity.

**Table 7:** Covariate-Adjusted R3STEP results across pairwise profile comparisons.

Comparison	B	S.E.	p-value	OR
P1 vs. P3	0.284	0.052	< 0.001	1.328
P2 vs. P3	0.383	0.036	< 0.001	1.466
P2 vs. P1	0.099	0.045	0.027	1.104

P1, low campus connectedness profile; P2, moderate campus connectedness profile; P3, high campus connectedness profile. B, logit regression coefficient; S.E., standard error; OR, odds ratio. The model was adjusted for sex, age, institution type, academic year, and monthly household income.

### 3.6 Differences in DER across campus connectedness profiles

Tables 8, 9 present differences in DER scores across the three campus connectedness profiles. As shown in Table 8, P1 had the highest mean DER score (*M* = 3.706, SE = 0.075), followed by P2 (M = 3.374, SE = 0.026), whereas P3 had the lowest mean DER score (M = 2.522, SE = 0.036). As shown in Table 9, the BCH test further showed a significant overall difference in DER scores across the three profiles. Pairwise comparisons indicated that the differences between P1 and P2, P1 and P3, and P2 and P3 were all statistically significant, with each comparison reaching *p* < 0.001.

**Table 8:** DER means of campus connectedness profiles.

Profile	Mean	s.e.
P 1	3.706	0.075
P 2	3.374	0.026
P 3	2.522	0.036

P1-P3, 3 Profiles of Campus Connectedness; S.E., Standard Error.

**Table 9:** BCH test for differences in DER scores across profiles.

Comparison	Chi-Square	p-Value
Overall test	423.747	< 0.001
P 1 vs. P 2	17.12	< 0.001
P 1 vs. P 3	201.989	< 0.001
P 2 vs. P 3	354.599	< 0.001

BCH, Bolck-Croon-Hagenaars; P1-P3, 3 Profiles of Campus Connectedness; DER, Difficulties in Emotion Regulation.

## 4 Discussion

The present study identified three ordered profiles of low, moderate, and high campus connectedness. The three-profile solution was supported by cross-solution comparisons and was broadly reproducible across sex. At the same assessment occasion, higher perceived power distance was associated with greater odds of membership in either of the two lower-connectedness profiles, relative to the high-connectedness profile. DER levels were progressively lower from the low- to the high-connectedness profile.

First, consistent with prior person-centered research on social connectedness (Rose et al., 2019; Teas et al., 2023; Oberle et al., 2024), the retained three-profile solution was characterized by low, moderate, and high levels of campus connectedness. However, the heterogeneity observed in the present study was primarily quantitative rather than configurational, as the profiles differed mainly in their overall levels across the indicators. One plausible explanation concerns the scope and operationalization of the construct. Broader conceptualizations of social connectedness may encompass multiple relational domains and sources of connection, potentially allowing individuals to display qualitatively different combinations of strengths and weaknesses (Rose et al., 2019; Teas et al., 2023; Oberle et al., 2024). In contrast, campus connectedness primarily refers to social belonging on campus rather than as a set of independently varying components, and was assessed here using a unidimensional scale which express an overall evaluation of one's place in the campus community (Lee and Robbins, 1995; Lee and Yoo, 2002; Allen et al., 2021; Dias-Broens et al., 2024). This may have produced profiles that varied mainly in elevation rather than shape. The cross-solution evidence further supported this predominantly level-based interpretation, and sex-stratified analyses suggested that the same general structure was broadly reproducible across sex. However, at the indicator level, Item 10 showed a modestly more pronounced relative decline in the lower-connectedness profiles. Whether this pattern reflects a distinctive role of close peer bonds or item-specific measurement properties cannot be determined from the present data and requires replication using multidimensional connectedness measures.

The second major finding was an asymmetric concurrent association between perceived teacher–student power distance and campus connectedness profile membership. At the same assessment occasion, higher perceived power distance was associated with greater odds of membership in either lower-connectedness profile relative to the high-connectedness profile, whereas the low–moderate contrast was weaker and model-dependent. Accordingly, the term asymmetric association is used here to refer to heterogeneity in association strength across profile contrasts, rather than associations in opposite directions. For college students, universities and colleges are constituted not only by horizontal peer networks (Sollitto et al., 2013; Wilson and Gore, 2013; Thelamour et al., 2019), but also by salient vertical hierarchical structures (Dai and Matthews, 2023; Matthews and Dollinger, 2023), in which teachers represent the most proximal embodiment of institutional authority (Hagenauer and Volet, 2014; Xu and Zhang, 2025). Lower perceived power distance may co-occur with perceptions of greater teacher approachability and more accessible institutional support (Kirby and Thomas, 2022; Abdulhamed and Beattie, 2024; Kim and Lundberg, 2026). Such perceptions would be compatible with stronger campus connectedness. Conversely, evidence concerning the low–moderate contrast was substantially weaker. Perceived power distance was not significantly associated with P2-vs.-P1 membership in the unadjusted model, and the significant association observed after covariate adjustment was quite small, inconsistent with a simple ordered-gradient pattern. The low–moderate contrast should therefore not be interpreted as a substantively robust profile difference. One possible explanation is that, in Chinese higher education, cultural norms and institutional arrangements may provide a context in which hierarchical teacher–student relations, and a certain degree of power distance, are socially expected and institutionally normalized (Dai and Matthews, 2023; Matthews and Dollinger, 2023). Against this background, perceived power distance may show relatively little differentiation between the low- and moderate-connectedness profiles. Meanwhile, differences between these two profiles may depend more strongly on other aspects of campus life, such as peer intimacy, campus participation, or person–environment fit, which are not uniquely captured by perceived teacher–student power distance. Overall, the results did not support a uniformly graded association across the three ordered profiles. Notably, perceived legitimacy of hierarchy, cultural norm endorsement, the accessibility of institutional support, and other proposed explanatory factors were not directly assessed in this study. These accounts should therefore be regarded as theoretically informed hypotheses rather than established explanations.

The third major finding concerned DER. DER scores differed significantly across the three campus connectedness profiles, exhibiting a monotonic decrease from P1 through P2 to P3, with all pairwise comparisons attaining statistical significance. These results lend profile-level external validity to the three-profile solution and align with prior variable-centered findings linking social connectedness to better emotional functioning (Xu et al., 2024; Huang et al., 2025). A plausible explanation is that students in the low-connectedness profile may be more uncertain about their standing on campus and more likely to interpret everyday setbacks as evidence of not belonging (Walton and Cohen, 2007), which may increase the emotional demands they need to regulate, while having fewer interpersonal channels for regulating them, such as disclosure, reassurance, or the reappraisal of stressful events within supportive interaction (Zaki and Williams, 2013; Liu et al., 2021). Heavier demands and thinner external resources may leave self-regulation more strained, constraining the regulatory basis of resilient adjustment (Polizzi and Lynn, 2021; Stover et al., 2024). Beyond this overall level-based pattern, the modestly sharper decline of close peer-bond perceptions (Item 10) in the lower profiles raises the tentative possibility that deeper peer intimacy may play a particular role in emotional support (Costello et al., 2023; Ruan et al., 2024). Importantly, this hypothesis requires direct testing in future studies with dedicated peer-intimacy measures, as the present design does not allow us to isolate which specific aspects of campus connectedness most strongly drive DER differences.

The theoretical contribution of this study lies in five respects. First, this study provides the first direct person-centered test of whether campus connectedness is organized as ordered level-based profiles or as qualitatively distinct configurational subtypes. The findings support a predominantly level-based structure under the present unidimensional operationalization and provide convergent person-centered support for variable-centered approaches to campus connectedness, which focus primarily on its overall levels. Second, the study identifies a modestly sharper relative decline in close peer-bond perceptions in the lower profiles as a single exploratory shape feature that warrants further investigation rather than a definitive marker of layered relational depth. Third, the profile-level analysis showed that the asymmetric association between perceived teacher–student power distance and campus connectedness varied across specific profile contrasts, with larger associations for contrasts involving the high-connectedness profile than for the low–moderate contrast. This profile-based contrast complements the continuous-score approach, which is less able to identify the specific levels at which associations with external variables are strongest, even when nonlinear associations are modeled (e.g., using quadratic terms). Fourth, a further contribution concerns the cultural boundary of belonging and connectedness frameworks. The present findings emerged within a Confucian-heritage context where teacher-student power distance is culturally normative and structurally salient, a condition underrepresented in the predominantly Western connectedness literature, although this cultural contingency remains a hypothesis rather than an established mechanism. Fifth, the monotonic decrease in difficulties in emotion regulation across the three ordered profiles provides external validity for the retained profile solution, indicating that this model-based representation of variation in campus connectedness corresponds to meaningful differences in psychological functioning relevant to resilient adjustment.

These findings carry implications for faculty development and campus support. Since profiles differed mainly in overall connectedness level rather than distinct configurations, a proportionate, tiered support model is warranted. Universal opportunities for peer and faculty contact through seminars, mentoring, small group learning, and student organizations should be available to all students, while proactive outreach and facilitated access to resources should target persistently low connectedness students. Brief, non-diagnostic connectedness screenings may be more practical than formal profile classification. The link between perceived teacher-student power distance and lower connectedness profiles suggests faculty training should not eliminate legitimate role differentiation but should instead reduce the relational costs of hierarchy. This can be achieved through transparent expectations, inviting questions and disagreement, autonomy-supportive language, predictable consultation opportunities, constructive responses to help-seeking, and appropriate referrals. Such training may best be delivered at the institutional or faculty-wide level, supplemented by targeted outreach to lower connectedness students. The monotonic decline in emotion regulation difficulties across profiles supports integrating relational and mental health support, combining peer support with emotion regulation programming for students showing both low connectedness and high difficulty. The sharper decline in close peer bonds points to repeated, deeper peer interaction opportunities, rather than one-time events, though this remains exploratory. Longitudinal or intervention studies should test these practices' effects on connectedness, regulation, help seeking, and profile transitions.

Several limitations should be noted. First, although the cross-sectional design allowed us to identify associations among perceived teacher–student power distance, campus connectedness profiles, and DER, it cannot establish temporal precedence or causal direction among these constructs. Future research could use longitudinal designs, latent transition analysis, or intervention-based approaches to examine how students move across connectedness profiles over time. Second, convenience and snowball sampling produced a female-majority sample (72.3%), limiting generalizability. Although sex-stratified analyses reproduced the ordered three-profile structure in both subsamples, weaker correspondence for the low-connectedness profile and descriptive prevalence differences indicated incomplete cross-sex equivalence. These analyses neither eliminate selection bias nor constitute formal multigroup invariance testing. Future studies should replicate the findings using sex-balanced probability samples and multi group mixture models. Third, the study was conducted within Chinese higher education, where teacher–student hierarchy may be culturally and institutionally salient. Thus, the findings should be interpreted within this specific educational context. In future studies, cross-cultural comparisons spanning Confucian-heritage and lower power-distance higher-education contexts would clarify whether this asymmetry is culturally bounded. Fourth, the study relied on students' self-reported perceptions. Although such perceptions are central to campus connectedness, this approach cannot fully distinguish perceived hierarchy from actual teacher behaviors, institutional practices, or interactional patterns. Future research could incorporate teacher reports, classroom observations, interviews, or behavioral indicators of teacher–student interaction. Fifth, the differentiation between the low and moderate connectedness profiles by perceived power distance was not robust. It was non-significant in the unadjusted model and attained significance only after covariate adjustment, with a small odds-ratio contrast and a direction inconsistent with a simple ordered-gradient pattern. This weak distinction should therefore be interpreted with caution, and future studies with larger, independent samples and finer-grained relational indicators are needed to verify whether, and under what conditions, perceived power distance is reliably associated with differential odds of membership in the two lower-connectedness profiles. Finally, the identified profiles mainly reflected ordered differences in the overall level of campus connectedness, rather than fully distinct configurations. This may partly reflect the unidimensional nature of the Campus Connectedness Scale. Future work could use multidimensional indicators, such as peer intimacy, faculty support, institutional identification, psychological safety, and campus participation, to identify more complex profiles of campus connectedness.

## 5 Conclusion

This study examined campus connectedness as a campus-specific relational expression of social belonging among Chinese college students and a three-profile solution characterized by ordered low, moderate, and high levels of connectedness was retained, with the profiles differing predominantly in elevation rather than representing qualitatively distinct configurational subtypes. The ordered structure was broadly reproduced across male and female participants, although modest differences in profile-shape correspondence and profile prevalence precluded claims of full cross-sex equivalence. A single close peer-bond item showed a modestly sharper relative decline in the lower profiles, offering preliminary and exploratory evidence that deeper peer intimacy may be somewhat more sensitive to lower overall connectedness than broader aspects of campus belonging. Perceived teacher–student power distance was concurrently associated with campus connectedness profile membership, and the pairwise contrasts showed an asymmetric rather than uniformly ordered pattern. Higher perceived power distance was associated with greater odds of membership in the low- or moderate-connectedness profile, relative to the high-connectedness profile, whereas the low-vs.-moderate association was comparatively small and model-dependent. This pattern may be consistent with the cultural and institutional salience of teacher–student hierarchy in Chinese higher education, although this interpretation was not directly tested and requires longitudinal and cross-cultural examination. DER levels decreased monotonically across the three profiles, providing external validity for the retained profile solution and indicating that this level-based representation of campus connectedness co-occurred with meaningful differences in emotional functioning relevant to adaptive and resilient adjustment. Taken together, the findings support a parsimonious, level-based person-centered account of campus connectedness as a campus-specific expression of social belonging, with systematic profile-level differences in DER further supporting the psychological relevance of this representation, while highlighting peer-bond depth and perceived institutional hierarchy as promising directions for future profile-level research.

## Data Availability

The raw data supporting the conclusions of this article will be made available by the authors, without undue reservation.
