# Supplementary material for: Perceived teacher–student power distance in relation to campus connectedness profiles among Chinese college students: profile differences in difficulties in emotion regulation
Source: Front Psychol. 2026 Aug 27;17:1906678. doi: 10.3389/fpsyg.2026.1906678 (PMC13557990; doi:10.3389/fpsyg.2026.1906678)
Supplement: Supplementary file 1 [file Table_1.docx]

**S1. Relative Standing of Item 10 Across Campus Connectedness Profiles**

Table S1 presents the within-profile relative standing of Item 10. The gap between the average of the remaining 13 CCS items and Item 10 increased from the high profile (.18) to the moderate profile (.57) and the low profile (.82). Pairwise comparison showed that adjacent gap differences were significant, with the moderate profile showing a larger gap than the high profile and the low profile showing a larger gap than the moderate profile, both p < .001. This pattern suggests that Item 10 became relatively lower within the CCS item profile as overall campus connectedness decreased.

**Table S1. Relative Standing of Item 10 within Campus Connectedness Profiles**

| **Profile** | **Other** **item mean** | **Item 10 mean** | **Gap** | **Gap comparison** |
| --- | --- | --- | --- | --- |
| Profile 3 | 6.06 | 5.88 | .18 | Reference |
| Profile 2 | 4.59 | 4.02 | .57 | Profile 2 > Profile 3, p < .001 |
| Profile 1 | 2.77 | 1.95 | .82 | Profile 1 > Profile 2, p < .001 |

Note: Profile 3, High campus connectedness profile; Profile 2, Moderate campus connectedness profile; Profile 1, Low campus connectedness profile; Other item mean, the average of the remaining 13 Campus Connectedness Scale items; Gap, the average of the remaining 13 Campus Connectedness Scale items minus Item 10. Gap was calculated as the other 13 items minus Item 10. All adjacent gap comparisons were significant at p < .001.
