# Supplementary material for: Perceived teacher–student power distance in relation to campus connectedness profiles among Chinese college students: profile differences in difficulties in emotion regulation
Source: Front Psychol. 2026 Aug 27;17:1906678. doi: 10.3389/fpsyg.2026.1906678 (PMC13557990; doi:10.3389/fpsyg.2026.1906678)
Supplement: Supplementary file 2 [file Table_2.docx]

**S2. Sex-Stratified Latent Profile Analyses of Campus Connectedness**

**S2.1. Latent Profiles of Campus Connectedness** **in the Female Subsample**

As shown in Table S2, the AIC, BIC, and sample-size-adjusted BIC decreased progressively as the number of profiles increased, and both the LMRT and BLRT remained statistically significant across the competing solutions. However, the three-profile solution yielded the highest entropy value (.945), indicating the greatest classification precision among the estimated models. Moreover, the smallest profiles in the four- and five-profile solutions accounted for only 4.7% and 4.2% of the female subsample, respectively, raising concerns about their substantive stability and interpretability. The incremental reductions in the information criteria also became markedly smaller beyond the three-profile solution. Considering statistical fit, classification precision, profile size, interpretability, and parsimony, the three-profile solution was retained for the female subsample, comprising ordered low-, moderate-, and high-connectedness profiles.

**Table S2. Model fit indices for profile solutions in the Female Subsample**

| **PN** | **AIC** | **ΔAIC** | **BIC** | **ΔBIC** | **aBIC** | **ΔaBIC** | **ENTROPY** | **LMRT** | **BLRT** | **PMG** |
| --- | --- | --- | --- | --- | --- | --- | --- | --- | --- | --- |
| P1 | 136181.2 | —— | 136347.5 | —— | 136258.6 | —— | —— | —— | —— | —— |
| P2 | 120808.0 | 15373.2 | 121063.5 | 15284.1 | 120926.8 | 15331.7 | .924 | <.001 | <.001 | 43.1% |
| **P3** | **114536.8** | **6271.2** | **114881.4** | **6182.1** | **114697.1** | **6229.8** | **.945** | **.002** | **<.001** | **9.4%^a^** |
| P4 | 110937.4 | 3599.4 | 111371.1 | 3510.3 | 111139.2 | 3557.9 | .922 | <.001 | <.001 | 4.7% |
| P5 | 109242.3 | 1695.1 | 109765.1 | 1606.0 | 109485.5 | 1653.7 | .921 | <.001 | <.001 | 4.2% |

Note: PN, Profile Number; AIC, Akaike Information Criterion; BIC, Bayesian Information Criterion; aBIC, Sample-size-adjusted BIC; LMRT, Lo-Mendell-Rubin Adjusted Likelihood Ratio Test; BLRT, Bootstrapped Likelihood Ratio Test; PMG, Proportion of Minimum Group.

^a^ The best fit solution is indicated in bold.

As shown in Figure S1, the three-profile solution in the female subsample reflected primarily ordered differences in overall campus connectedness rather than qualitatively distinct configurations. Profile 1 showed consistently low standardized scores across all indicators and was labeled the low-connectedness profile. Profile 2 exhibited scores near, but generally below, the subsample mean and was labeled the moderate-connectedness profile. Profile 3 displayed uniformly positive scores and was labeled the high-connectedness profile. The broadly parallel trajectories indicate a predominantly level-based structure, although the relatively sharper decline on CC10 suggests limited item-specific variation within the overall pattern.

**Figure S1. Standardized Campus Connectedness Item Means in the Female Subsample.** Class-specific item means were standardized using the corresponding item means and standard deviations in the Female Subsample.

**S2.2. Latent Profiles of Campus Connectedness in the Male Subsample**

As shown in Table S3, the AIC, BIC, and aBIC decreased as the number of profiles increased. The three-profile solution substantially improved on the two-profile solution, with marked reductions in all three information criteria and higher entropy (.965 vs. .948). According, the two-profile solution was not retained. Thereafter, Figure S2 showed diminishing gains, with progressively smaller decreases from three to four, four to five, and five to six profiles. Although the LMRT and BLRT were significant through the five-profile solution, the six-profile solution produced the smallest improvement, a nonsignificant LMRT (p = .478), and a smallest profile comprising only 3.1% of the sample, raising stability concerns. The six-profile solution was therefore excluded from further consideration. Among the remaining solutions, the three-profile model had the highest entropy (.965) and a larger minimum profile proportion (8.9%) than the four- and five-profile models. Balancing statistical fit, classification accuracy, parsimony, and profile size, the three-profile solution was selected.

**Table S3. Model fit indices for profile solutions in the Male Subsample**

| **PN** | **AIC** | **ΔAIC** | **BIC** | **ΔBIC** | **aBIC** | **ΔaBIC** | **ENTROPY** | **LMRT** | **BLRT** | **PMG** |
| --- | --- | --- | --- | --- | --- | --- | --- | --- | --- | --- |
| P1 | 54617.8 | —— | 54757.2 | —— | 54668.3 | —— | —— | —— | —— | —— |
| P2 | 47529.6 | 7088.132 | 47743.8 | 7013.417 | 47607.2 | 7061.1 | .948 | <.001 | <.001 | 33.0% |
| **P3** | **44022.5** | **3507.165** | **44311.4** | **3432.45** | **44127.1** | **3480.1** | **.965** | <.001 | <.001 | **8.9%** |
| P4 | 42586.6 | 1435.872 | 42950.2 | 1361.157 | 42718.3 | 1408.8 | .944 | <.001 | <.001 | 6.8% |
| P5 | 41750.7 | 835.9 | 42189.1 | 761.1 | 41909.6 | 808.8 | .938 | <.001 | <.001 | 6.8% |
| P6 | 41512.0 | 238.7 | 42025.0 | 164.1 | 41697.9 | 211.7 | .946 | .478 | <.001 | 3.1% |

Note: PN, Profile Number; AIC, Akaike Information Criterion; BIC, Bayesian Information Criterion; aBIC, Sample-size-adjusted BIC; LMRT, Lo-Mendell-Rubin Adjusted Likelihood Ratio Test; BLRT, Bootstrapped Likelihood Ratio Test; PMG, Proportion of Minimum Group.

^a^ The best fit solution is indicated in bold.

**Figure S2. Changes in Information Criteria Indices Across Profile Solutions in the Male Subsample**

The mean-centered Tucker’s congruence coefficients reported in Panels A and B of Table S4 provided further support for the three-profile solution. In the four-profile solution shown in Panel A, the additional profile appeared to reflect a subdivision of the moderate-connectedness profile identified in the three-profile solution. A similar pattern was observed in the five-profile solution shown in Panel B, in which the additional profiles largely represented further differentiation of profiles already present in the three-profile solution. Although one profile in the five-profile solution showed comparatively weaker correspondence with the three original profiles, inspection of the standardized profile plots in Figure S3 indicated that the three-, four-, and five-profile solutions were all characterized primarily by differences in overall elevation rather than by qualitatively distinct configurations. Consequently, the additional profiles did not provide substantively meaningful or theoretically incremental patterns of campus connectedness. Taken together, the diminishing gains in model fit, highest entropy, adequate profile sizes, cross-solution congruence, and absence of theoretically distinct additional profiles supported retention of the more parsimonious three-profile solution.

**Table S4. Centered Tucker’s Congruence Coefficients and Profile Elevations across the Three-, Four-, and Five-Profile Solutions in the Male Subsample.**

Panel A. Three- and Four-Profile Solutions

| **Profile** | **4P-1** | **4P-2** | **4P-3** | **4P-4** | **3P Elevation** |
| --- | --- | --- | --- | --- | --- |
| **3P-1** | **.962** | .816 | .621 | .138 | -1.705 |
| **3P-2** | .692 | **.953** | **.983** | .655 | -.322 |
| **3P-3** | .172 | .601 | .827 | **.984** | .742 |
| **4P Elevation** | -1.873 | -.718 | .006 | .822 | —— |

Panel B. Three- and Five-Profile Solutions

| **Profile** | **5P-1** | **5P-2** | **5P-3** | **5P-4** | **5P-5** | **3P Elevation** |
| --- | --- | --- | --- | --- | --- | --- |
| **3P-1** | **.950** | .814 | .657 | .379 | -.278 | -1.705 |
| **3P-2** | .670 | **.949** | **.989** | .833 | .275 | -.322 |
| **3P-3** | .136 | .596 | .800 | **.982** | **.806** | .742 |
| **5P Elevation** | -1.881 | -.745 | -.074 | .555 | 1.175 | —— |

Note: Congruence coefficient entries are centered Tucker’s congruence coefficients calculated from within-profile mean-centered standardized item means in the Male Subsample. 3P, 4P, and 5P denote the three-, four-, and five-profile solutions, respectively, and the number following the hyphen identifies the profile within each solution. Profile elevation was calculated as the mean of the 14 standardized item means within each profile. Profiles within each solution are ordered by increasing elevation. Boldface indicates the strongest correspondence for each profile in the four- or five-profile solution.

(A)

(B)

(C)

**Figure S3. Standardized Campus Connectedness Item Means Across the Three-, Four-, and Five-Profile Solutions in the Male Subsample.** Panels A, B, and C present the three-profile, four-profile, and five-profile solutions, respectively. Class-specific item means were standardized using the corresponding item means and standard deviations in the male Subsample.

**S2.3. Cross-Sex Similarity of the Three-Profile Solution**

As shown in Table S5, both subsamples exhibited the same ordered low-, moderate-, and high-connectedness structure, with broadly comparable profile elevations. The centered Tucker’s congruence coefficients for the corresponding low-, moderate-, and high-connectedness profiles were .760, .939, and .869, respectively. Thus, cross-sex shape similarity was strongest for the moderate-connectedness profile, acceptable for the high-connectedness profile, and comparatively weaker for the low-connectedness profile. Moreover, the male moderate-connectedness profile showed relatively high congruence with all three female profiles, consistent with the predominantly level-based and broadly parallel nature of the profiles. The profile proportions also exhibited the same rank ordering across sex, with the moderate-connectedness profile being the most prevalent, followed by the high- and low-connectedness profiles. More specifically, the prevalence of the low-connectedness profile was nearly identical across sex, whereas the moderate-connectedness profile was descriptively more prevalent among female participants and the high-connectedness profile was more prevalent among male participants. Taken together, the results support the broad cross-sex reproducibility of the ordered three-profile structure, while also indicating modest cross-sex differences in profile-shape correspondence and profile prevalence.

**Table S5. Centered Tucker’s Congruence Coefficients, Profile Elevations, and Profile Proportions Across the Male and Female Three-Profile Solutions**

| **Profiles** | **Female low** | **Female moderate** | **Female high** | **Male elevation** | **Male proposition** |
| --- | --- | --- | --- | --- | --- |
| **Male low** | .760 | .629 | .373 | -1.692 | 8.92% |
| **Male moderate** | .908 | .939 | .897 | -.251 | 49.16% |
| **Male high** | .489 | .603 | .869 | .857 | 41.91% |
| **Female elevation** | -1.480 | -.276 | .733 | — | — |
| **Female proposition** | 9.36% | 55.14% | 35.49% | — | — |
