# Supplementary material for: Perceived teacher–student power distance in relation to campus connectedness profiles among Chinese college students: profile differences in difficulties in emotion regulation
Source: Front Psychol. 2026 Aug 27;17:1906678. doi: 10.3389/fpsyg.2026.1906678 (PMC13557990; doi:10.3389/fpsyg.2026.1906678)
Supplement: Supplementary file 3 [file Table_3.docx]

**S3. Complementary Continuous-Score Analysis**

A complementary continuous-score analysis was conducted to examine whether the concurrent association between perceived teacher–student power distance and campus connectedness was adequately characterized by a linear relationship. In the linear model, higher perceived power distance was associated with lower campus connectedness (B = −0.150, SE = 0.013, p < .001), with the model accounting for 3.1% of the variance in campus connectedness (R² = .031). Adding the quadratic term significantly increased the explained variance by 10.4% (ΔR² = .104), resulting in a total adjusted R² of .135. In the quadratic model, both the linear term (B = −0.191, SE = 0.013, p < .001) and the quadratic term (B = 0.154, SE = 0.007, p < .001) were statistically significant. These findings indicated that the concurrent association between perceived power distance and campus connectedness departed substantially from a simple linear form. Full regression coefficients are reported in Supplementary Table S6.

**Supplementary Table S6. Linear and quadratic associations between perceived teacher–student power distance and campus connectedness**

| **Model** | **Variable** | **B** | **SE** | **β** | **t** | **p** | **R²** | **Adjusted R²** | **ΔR²** |
| --- | --- | --- | --- | --- | --- | --- | --- | --- | --- |
| Linear | PD | −0.150 | 0.013 | −.177 | −11.229 | < .001 | .031 | .031 | — |
| Quadratic | PD | −0.191 | 0.013 | −.226 | −14.971 | < .001 | .136 | .135 | .104 |
|  | PD² | 0.154 | 0.007 | .326 | 21.634 | < .001 |  |  |  |

Note: PD, perceived teacher–student power distance; CC, campus connectedness. CC was represented by the mean score of the 14-item Campus Connectedness Scale. PD was represented by the mean scale score and was mean-centered before the linear and quadratic terms were calculated; PD² denotes the squared mean-centered PD term. B, unstandardized regression coefficient; SE, standard error; β, standardized regression coefficient.
